# Advancing successful implementation of task-shifted mental health care in low-resource settings (BASIC): protocol for a stepped wedge cluster randomized trial

**DOI:** 10.1186/s12888-019-2364-4

**Published:** 2020-01-08

**Authors:** Shannon Dorsey, Christine L. Gray, Augustine I. Wasonga, Cyrilla Amanya, Bryan J. Weiner, C. Micha Belden, Prerna Martin, Rosemary D. Meza, Andrew K. Weinhold, Caroline Soi, Laura K. Murray, Leah Lucid, Elizabeth L. Turner, Robyn Mildon, Kathryn Whetten

**Affiliations:** 1 Department of Psychology, University of Washington Guthrie Hall 119A, Box 351525, Seattle, WA 98195 USA; 2 Center for Health Policy and Inequalities Research, Duke Global Health Institute, Duke University, Campus Box 90392, Durham, NC 27710 USA; 3 Research Department, Ace Africa Kenya, P.O. Box 1185, Bungoma, 50200 Kenya; 4 Department of Global Health, University of Washington, Harris Hydraulics Laboratory, 1510 San Juan Road, Seattle, WA 98195 USA; 5 Department of Health Services, School of Public Health, University of Washington, Box 357965, Seattle, WA 98195 USA; 6 Department of Mental Health, Johns Hopkins Bloomberg School of Public Health, 624 N. Broadway, 8th floor, Baltimore, MD 21205 USA; 7 Department of Biostatistics and Bioinformatics, Duke University School of Medicine, Duke University, Durham, NC 27710 USA; 8 Duke Global Health Institute, Duke University, Campus Box 90519, Durham, NC 27708 USA; 9 Centre for Evidence and Implementation, 33 Lincoln Square South, Carlton, Victoria 3053 Australia; 10 Terry Sanford Institute of Public Policy, Duke University, Box 90239, Durham, NC 27708 USA

**Keywords:** Global mental health, Implementation science, Evidence-based treatment, Task-shifting, Task-sharing, Children, Adolescents, School-based mental health care, Organizational theory, Implementation climate

## Abstract

**Background:**

The mental health treatment gap—the difference between those with mental health need and those who receive treatment—is high in low- and middle-income countries. Task-shifting has been used to address the shortage of mental health professionals, with a growing body of research demonstrating the effectiveness of mental health interventions delivered through task-shifting. However, very little research has focused on how to embed, support, and sustain task-shifting in government-funded systems with potential for scale up. The goal of the *Building and Sustaining Interventions for Children (BASIC)* study is to examine implementation policies and practices that predict adoption, fidelity, and sustainment of a mental health intervention in the education sector via teacher delivery and the health sector via community health volunteer delivery.

**Methods:**

BASIC is a Hybrid Type II Implementation-Effectiveness trial. The study design is a stepped wedge, cluster randomized trial involving 7 sequences of 40 schools and 40 communities surrounding the schools. Enrollment consists of 120 teachers, 120 community health volunteers, up to 80 site leaders, and up to 1280 youth and one of their primary guardians. The evidence-based mental health intervention is a locally adapted version of Trauma-focused Cognitive Behavioral Therapy, called *Pamoja Tunaweza.* Lay counselors are trained and supervised in *Pamoja Tunaweza* by local trainers who are experienced in delivering the intervention and who participated in a Train-the-Trainer model of skills transfer. After the first sequence completes implementation, in-depth interviews are conducted with initial implementing sites’ counselors and leaders. Findings are used to inform delivery of implementation facilitation for subsequent sequences’ sites. We use a mixed methods approach including qualitative comparative analysis to identify necessary and sufficient implementation policies and practices that predict 3 implementation outcomes of interest: adoption, fidelity, and sustainment. We also examine child mental health outcomes and cost of the intervention in both the education and health sectors.

**Discussion:**

The BASIC study will provide knowledge about how implementation of task-shifted mental health care can be supported in government systems that already serve children and adolescents. Knowledge about implementation policies and practices from BASIC can advance the science of implementation in low-resource contexts.

**Trial registration:**

Trial Registration: ClinicalTrials.gov Identifier: NCT03243396. Registered 9th August 2017, https://clinicaltrials.gov/ct2/show/NCT03243396

## Background

Eighty percent of the world’s population lives in low and middle-income countries (LMICs), yet very few of the world’s mental health resources are in LMICs. Mental health disorders are now estimated to be first (32.4%) in the global burden of disease for years lost to disability [1]. With low tax bases and high population needs, governments in LMICs spend less than 2% of their health budgets on mental health [2], with most resources targeting care for the adult, seriously mentally ill population. A critical need exists to identify strategies to address the mental health treatment gap for children in LMICs that can be scaled up without substantial new resources, yet limited implementation research has occurred in global mental health [3, 4].

One commonly used strategy for addressing the shortage of mental health professionals in LMICs and reducing the mental health treatment gap is task-shifting [5, 6]. Task-shifting involves using non-specialists or paraprofessionals (lay counselors) with little to no prior mental health training or experience to deliver care, under supervision. A growing body of research and a recent Cochrane review [7] provide evidence that task-shifted mental health care is an effective strategy to address the mental health treatment gap. Randomized controlled trials (RCTs) across culturally diverse LMICs (e.g., Zambia [8], Uganda [9, 10], India [11], Southern Iraq [12]) and populations of focus (e.g., adults, adolescents, displaced persons, rural areas) indicate that evidence-based treatments (EBTs) can be effectively delivered. Effect sizes for primary treatment outcomes are typically medium to large (with a range of comparison conditions). Some studies also have shown effectiveness for broader outcomes (e.g., infant health in rural Pakistan [13] when maternal depression was treated). Outcomes appear to be sustained in trials that included follow-up (e.g., 6–12 months) [13, 14]. Research indicates provider and client acceptability and satisfaction with EBTs [15–17]. These studies suggest that EBT delivery via task-shifting is acceptable and can be effective, although there are some concerns about feasibility [16], including the necessary ongoing support for lay counselors and their organizations to successfully implement mental health interventions.

The World Health Organization (WHO) suggests “beginning with the end in mind,” only considering solutions that might be candidates for scale-up and sustainability within the low-resource context (i.e., limited funding, few mental health professionals). Task-shifting is a promising strategy, however, very limited research has focused on how to embed, support, and effectively sustain EBTs via task-sharing in government-funded systems in which they could be scaled up [16]. The lack of research and knowledge in this area is a substantial barrier to bridging the mental health treatment gap and improving population health and wellbeing.

### Conceptual framework/approach

*“Building and Sustaining Interventions for Children (****BASIC****): Task Sharing Mental Health Care in Low-Resource Settings”* builds on our 15-year history of collaborations with Ace Africa in Kenya, and recent work evaluating the effectiveness of Trauma-focused Cognitive Behavioral Therapy (TF-CBT) [19] (“*Pamoja Tunaweza*”) with children who experienced parental death [20, 21] and have mental health impact. Our goal is to identify locally sustainable implementation policies and practices (IPPs) that lead to effective implementation of task-shifted EBT delivery (*Pamoja Tunaweza* in this study) in 2 governmental sectors in Kenya, identified by our Kenyan partners as potential platforms for scale-up—Education (via teacher delivery) and Health (via community health volunteer [CHV] delivery). Both Education and Health may be viable sectors for mental health care delivery, but the IPPs that predict implementation success and intervention effectiveness in either/both sectors are unknown. In this study, we identify contextually relevant, practical, and actionable IPPs that can inform implementation planning. We also assess child outcomes and intervention costs in both sectors.

In 2016, Betancourt and Chambers [4] proposed 5 specific areas to advance implementation science knowledge around mental health delivery in LMICs (see Table 1). Our past work addressed the first 2 areas: identifying treatment providers and addressing training and support. Informed by a theoretical model of effective training (the Training Transfer Conceptual Model [22–24]), we used implementation strategies [25] including making training dynamic, modeling and simulating change, and providing clinical supervision. BASIC extends this work by broadening attention to include other organizational factors that may influence lay counselor EBT delivery in either the Education or Health sector (latter 3 areas in Table 1).

**Table 1:** Specific areas to advance implementation science knowledge for mental health delivery in LMICs*

Who should deliver practices?	
How should they be trained and supported?	
How to sustain and adapt?	
Use of technology	
Cost per outcomes	

*Adapted from Betancourt and Chambers, 2016

**Fig. 1:**
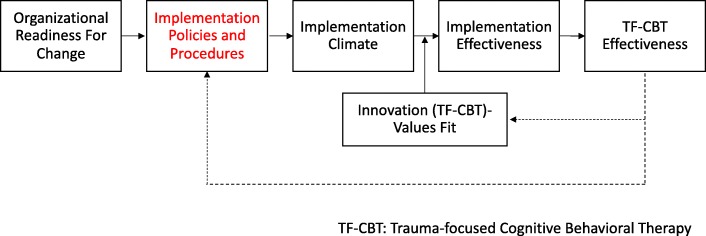
Determinants of Implementation Effectiveness* Legend: *Adapted from Weiner et al., 2009

We use Weiner and colleagues’ organizational theory of implementation effectiveness (Fig. 1), which posits that effective implementation (e.g., consistent, high-quality delivery of EBT) depends on the extent to which EBT deliverers experience a positive implementation climate, that is, a shared understanding that EBT delivery is expected, supported, and rewarded [26]. The extent to which implementation climate is positive depends, in turn, on the implementation policies and practices (IPPs) put into place to support EBT delivery. IPPs can include a wide range of structures and processes such as workload adjustment, resource provision, and rewards and/or incentives. The number, type, and strength of IPPs put into place depends on the organization’s readiness for change.

## Methods

### Trial design

**Fig. 2:**
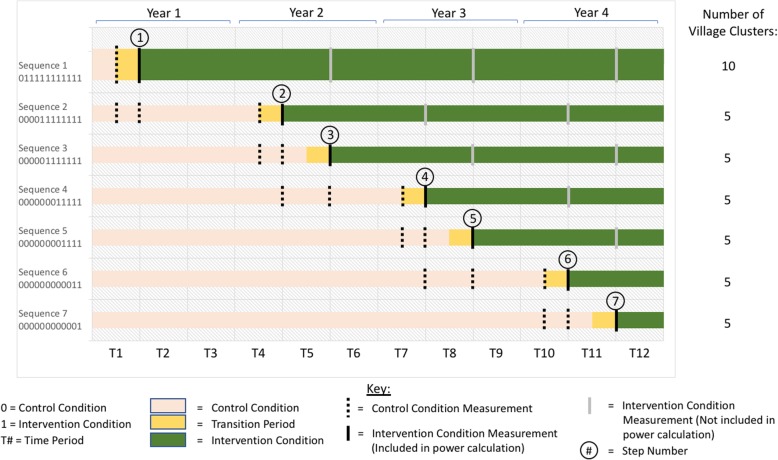
BASIC Stepped Wedge Design and Timeline Legend: Figure 2 depicts the overall study design of the incomplete stepped wedge cluster-randomized trial, including measurement time points for each sequence. A sequence is a group of clusters that initiate the intervention in the same time period. A step is the specific time point that participants receive the intervention and cross over from being treatment-naïve to having received treatment

We employ an incomplete stepped wedge, cluster randomized controlled trial (SW-CRT) design and mixed methods. For reporting, we follow the Consolidated Standards of Reporting Trials (CONSORT) for stepped wedge cluster-randomized trial designs [27] and the Standard Protocol Items: Recommendations for Interventional Trials (SPIRIT) guidelines (Additional file 1) [28]. We conduct a Hybrid Type II Implementation-Effectiveness trial [29] in both the Education and Health sectors in Bungoma South Sub-County in Kenya. All 137 primary schools in Bungoma South agreed to participate, and 40 schools were randomly selected. The surrounding community in which each school is nested is the health sector setting for the trial. The school and the surrounding community are considered a “village cluster.” Each of the 40 “village clusters” has 1 team of teachers and 1 team of CHVs delivering Pamoja Tunaweza (TF-CBT [19]). The CHV role in the Kenyan Health sector is that of extending health services from health facilities to communities, under the supervision of a community health extension worker (CHEW). We randomly order the 40 clusters and begin with 10 clusters in the first of 7 sequences of the SW-CRT (see Fig. 2), with 3 teachers and 3 CHVs in each cluster (totaling 240 providers) and up to 16 youth per sector per cluster (totaling 1280 youth) over the 7 sequences. The sample size of 40 schools and 40 surrounding communities is selected to allow for examining outcomes at the site/organization level. This sample size also provides sufficient numbers of counselors by type (teachers, CHVs) and youth so that we are sufficiently powered to answer study questions (see Aim 3; Power/Sample Size Calculation section).

### Overview of study aims

In Aim 1, we investigate TF-CBT implementation in the first sequence of the SW-CRT to identify actionable (i.e., modifiable) IPPs associated with successful implementation in schools and communities with different attributes (see Table 2). We then use these findings to inform implementation facilitation for subsequent sites (clusters that initiate intervention in sequences 2–7; 5 schools and communities per cluster) with BASIC collaborative teams (BCTs) comprised of Ace Africa staff and selected teachers/CHVs. To facilitate implementation, we leverage aspects of community development team models by Chamberlain [30] and Aarons [31] in the United States (US). In Aim 2, we test identified IPPs from the initial sequence, and other IPPs that emerge in subsequent sequences, as mechanisms of adoption and fidelity. We also examine IPPs that predict sustainment for the subset of sites (20 in each sector) that are followed for at least 2 years after the initial implementation year. In Aim 3, we examine TF-CBT effectiveness (child outcomes) and cost for each sector.

**Table 2:** Specific implementation policies and practices (IPPs)

Supervision participation	
Workload adjustment	
Resource provision	
Work flexibility	
Rewards/Incentives	

### Study setting

**Fig. 3:**
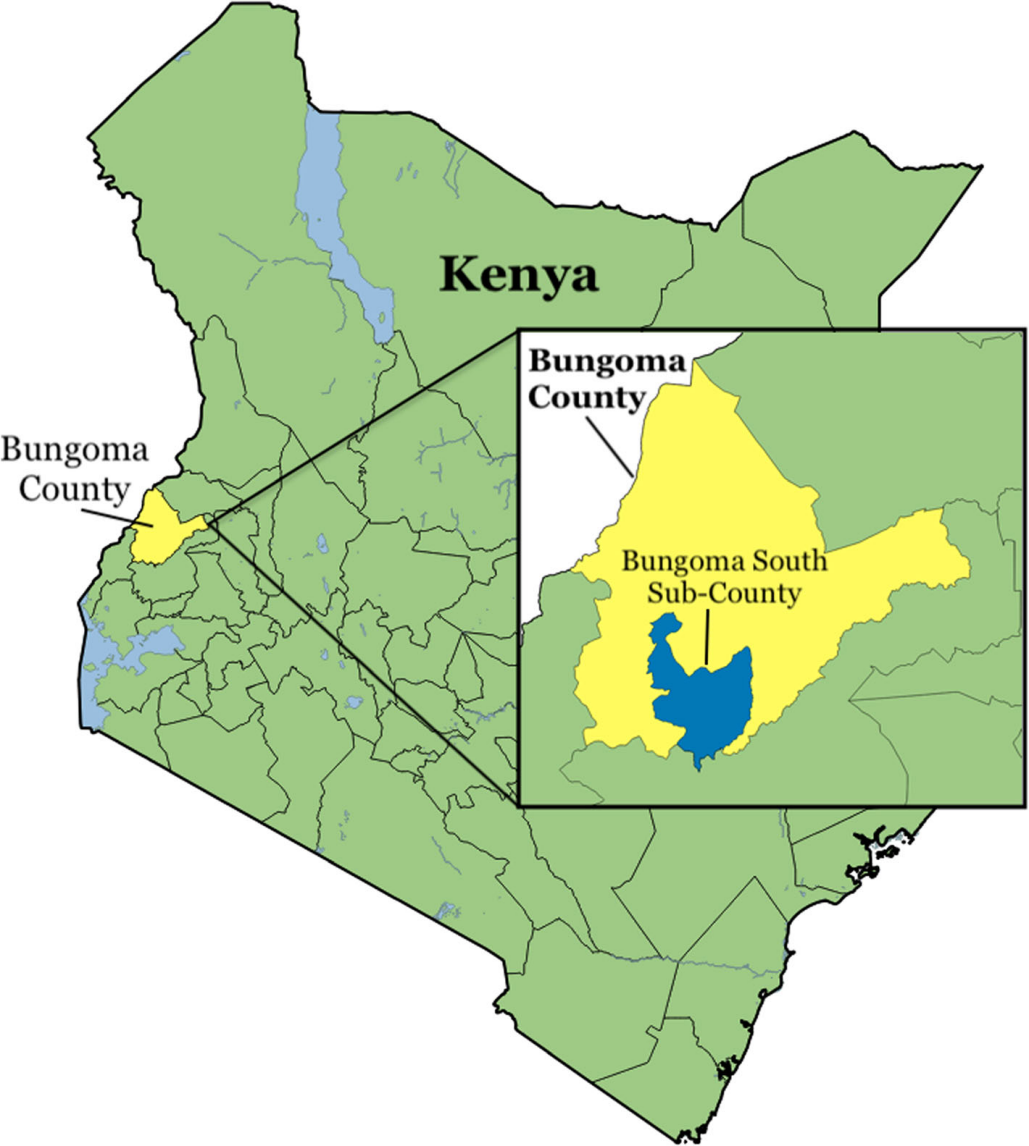
Bungoma South Sub-County Legend: Figure created using material from two sources: Boundaries: Updated November 2017 by Field Information and Coordination Support Section (FICSS), Division of Programme Support and Management (DPSM), UNHCR. https://data.humdata.org/dataset/ken-administrative-boundaries Water bodies: Added December 2007 by World Resources Institute (WRI), Nature’s Benefits in Kenya. https://www.wri.org/resources/data-sets/kenya-gis-data

In 2010 the Kenyan government began a decentralization process [32]. Kenya is divided into 47 counties that have significant political decision-making, organizational power, and funding. Kenya’s newly enacted National Mental Health Policy demonstrates a commitment to mental health [33–35]. Bungoma County is the third most populated county in Kenya with 1.7 million residents. Within Bungoma County, the study takes place in Bungoma South Sub-County (Fig. 3), which contains both urban and rural areas. Important for population health, nearly 50% of Bungoma County’s population are children < 15 years. Mental health professionals are largely unavailable. In the Bungoma Township, there are 2 psychiatric nurses and 1 psychiatrist.

### Intervention description

We selected TF-CBT, a child/adolescent EBT for psychosocial sequelae of trauma exposure (e.g., posttraumatic stress [PTS], depressive symptoms, anxiety), as it has the strongest empirical base [36, 37]. There are over 15 RCTs [37], including 1 in Zambia by team members[8]. A feasibility study in Tanzania demonstrated initial acceptability of TF-CBT for lay counselors, guardians, and children, with promising clinical outcomes [20]. In this feasibility study and the recently completed RCT in Tanzania and Kenya, The *Pamoja Tunaweza* intervention was 12 weeks in duration. Each week, 8 children and 1 of their guardians met concurrently and separately, with joint child-guardian activities in the final 4 sessions [20, 38]. TF-CBT was delivered via 3-counselor teams, with 2 counselors leading the child group, and 1 leading the guardian group. TF-CBT components (psychoeducation, parenting, relaxation, affective modulation, cognitive coping, in vivo exposure planning, and grief-specific skills) were delivered in groups, and 2 to 3 individual sessions mid-group were used for the imaginal exposure component (i.e., talking about/processing traumatic events).

#### Brief version of TF-CBT

More recent work (2015–16) involved testing a shortened 8-session version of TF-CBT (vs. 12 sessions) in Bungoma South, to be responsive to calls in implementation science and global health for greater intervention efficiency with the idea that simpler, shorter interventions [39] may be better candidates for scale-up, even if they come with a drop in effect size. This 8-session version, designed collaboratively with the Ace Africa counselors, includes all TF-CBT elements integrated into fewer sessions. A pilot study with 63 youth provided preliminary support for using the 8-session version in the BASIC trial, with BASIC providing a rigorous test.

### Participant eligibility and selection

#### Counselors and site leaders

Given the 3-counselor structure for group TF-CBT, and 40 sites for each sector, we enroll 120 teachers from Education and 120 CHVs from Health. Following an initial BASIC sensitization meeting with each site, Ace Africa works with site leadership to recruit 3 lay counselors. Sites begin delivering TF-CBT at different times, given the SW-CRT design. We recruit one leader at each site to participate in the study but not to deliver TF-CBT (i.e., head teachers and sometimes deputy teachers in schools; community health extension workers [CHEWs] in the health facility to which the CHVs are connected). Teachers who participate are selected based on being viewed as good with children experiencing difficulties, and ideally are not teachers of potential participants (to minimize any discomfort for children due to teaching and counseling overlap). Teachers deliver TF-CBT during the school week, in classrooms, during a 1-hour games time slot. CHVs are selected using similar criteria (i.e., good with children experiencing difficulties) and deliver TF-CBT at a time convenient for them. Delivery can occur in schools or other community spaces.

#### Youth and guardians

All single- (1-parent died) or double-orphaned children (both parents died; UNICEF definitions [40]) ages 11–14 in the area served by the identified school (n~ 40–50 per cluster) are enumerated in lists provided by both the schools and communities. The lists are then divided by sex. Within each sex, children are randomly ordered to be screened for PTS and prolonged grief using the same established brief screener and eligibility scores from the prior RCT. Children can screen in from child- or guardian-reported PTS symptoms or child-reported prolonged grief symptoms. TF-CBT groups are single sex, with up to 8 children per group. Once 16 children within a single sex screen in (or list has been exhausted), the study investigators use a computerized random number generator to randomize children to receive TF-CBT through Education or Health. The random assignment lists are then shared with the local interviewers who enroll and consent participants, and those interviewers call participants to inform participants of the group to which they have been randomly assigned, along with corresponding logistical details. In each of the 40 clusters, up to 32 children (16 girls; 16 boys) and 1 of their primary guardians receive the intervention.

Trained local interviewers obtain informed consent from all participants in person at the time of enrollment. For child participants, interviewers obtain both guardian permission and child assent.

### BASIC procedures

Once clusters within a sequence are scheduled to begin implementation, children are screened for TF-CBT eligibility. Teacher (embedded in the schools) and CHV teams (embedded in villages, via Health) are trained in TF-CBT and begin practice and supervision. They then provide 2 sequential 8-week TF-CBT groups (girls and boys, mapping onto 2 consecutive trimesters for schools; order of delivering girl or boy groups first alternates each calendar year). By the end of the study, this results in 40 sites per sector (80 total), 120 counselors per sector (240 total, given counselor teams of 3 in each site), and up to 640 children per sector (up to 1280 total) receiving TF-CBT (see Table 3).

**Table 3:** Participant Distribution

	Education	Health
Sites	40	40
Counselors	120	120
Groups	80	80
Youth	640	640

Prior to cross over, each cluster serves as its own control (allowing for within-cluster within-subject analyses) and as a control for clusters in the other sequences that receive intervention in the same time period (allowing for between-subject between-cluster analyses and to control for seasonal and other time effects). Counselors and leaders in each cluster participate in yearly assessments, as well as an assessment after they have delivered the 2 sequential TF-CBT groups (1 for each sex). Children participate in a baseline, end of treatment, and up to 3 annual post-treatment follow-up visits, depending on their sequence. The open cohort design allows counselors, children, and guardians enrolled in a period prior to the one in which they initiate their intervention to be replaced if not available or not eligible (e.g., child age > 14) when their cluster crosses over (i.e., TF-CBT starts) [33, 41].

#### Trainer training

Ace Africa has 5 experienced TF-CBT counselors from the RCT who serve as trainers and supervisors (termed “local trainers”) for counselors in schools and villages. This team co-developed and piloted the 8-session version of TF-CBT for group modality and parental-death focus. Ace Africa counselors participate in a Train-the-Trainer (TTT) training led by the first author. The TTT includes active and experiential strategies to facilitate adult learning and skill acquisition, following recommendations from reviews of provider training [42, 43]. Following the TTT, the local trainers have 2 months to refine training plans, practice training, and receive support. Local trainers receive weekly group supervision from TF-CBT experts on the study team during their first year in this role (1–1.5 hours per week, via Skype audio). In year 2 and forward, they continue to receive weekly support, but the focus is more on monitoring participant safety and logistics (i.e., monitoring implementation, applying the established safety protocol, and study timelines).

#### Counselor training and supervision

Following the Apprenticeship Model [44], local trainers provide 5–6 days of training for lay counselors (with separate trainings for teachers and CHVs). Using the same format of the TTT, lay counselors first receive didactic training and manuals for each session (i.e., “step sheets” for session delivery). Next, they participate in an experiential activity when relevant (e.g., trying cognitive coping), discuss benefits to build buy-in, read the steps one-by-one as a group, and then observe the trainer(s) modeling the skills while following along on the step sheets. Finally, trainees break into small groups and practice with both trainer and peer feedback.

#### Supervision/intervention quality assurance procedures

Following the in-person training, 1 local trainer begins weekly supervision with each counselor team. Supervision involves discussing the past session and role-playing upcoming sessions. Supervision is delivered via a mix of in-person and use of audio or video (e.g., phone call, review of videotaped role-play) with some support provided via text messaging, following remote supervision procedures from the RCT. Local trainers look for strong counselors who may be candidates for future TF-CBT leadership positions (e.g., future supervisors themselves, BASIC collaborative team members; see Aim 1 Procedures). In the sustainment phase, supervision occurs less frequently (e.g., 1 x/month), and is less frequently provided in-person.

#### Fidelity monitoring

Counselors complete a brief report for each session that includes child/guardian attendance, a self-report on fidelity to the session, and a brief note on participant response. Reports are reviewed by local trainers to inform supervision. Local trainers conduct live observation of groups 2x/month to both inform supervision and conduct fidelity monitoring during the initial implementation year and strive for 1x/month during sustainment.

### Data sources

#### Implementation policies and practices (IPPs)

For Aim 1/Sequence 1, IPPs are measured via quantitative measures and from qualitative interviews with counselors and leaders from selected sites participating in the case comparison analysis (see Aim 1). For Aim 2/Sequences 2–7, IPPs are measured using work plans and follow-up forms from implementation facilitation meetings (see Aim 2).

#### Primary study outcomes

The primary outcomes of interest are TF-CBT adoption, fidelity, and sustainment. Adoption is defined as delivery of 2 on-site 8-session TF-CBT groups by a 3-counselor team and is measured by counselor self-report (and confirmed by supervisors). Fidelity includes assessment of adherence and competency and is primarily measured by supervisors’ observations of TF-CBT groups using 1–6 ratings on adherence and 1–6 ratings on competence. Each week, even when live observation does not occur, the supervisor completes the same adherence and fidelity ratings (1–6) based on review of counselor reports and supervision interactions with the counselors. Based on studies that examined sustainment of other health interventions in African countries [41] and sustainment reviews [45, 46], we define sustainment as maintained delivery 2 years after the study intervention period (2 groups delivered within a calendar year, with at least 80% capacity as compared to their group enrollment during initial implementation).

#### Secondary study outcomes

We use brief, validated measures from our prior work in Kenya (already translated, back-translated, vetted for appropriateness/understanding, and Institutional Review Board (IRB)-approved [Duke Health & Kenya IRBs] to assess children’s mental health from both child and guardian perspective (e.g., trauma exposure, PTS, prolonged grief), and other relevant outcomes and constructs (e.g., strengths and difficulties, substance use, child-guardian relationship, and social support). Also included are several measures to address the priorities of interest to Kenyan policy makers due to value and/or high societal cost (e.g., orphan stigma, excessive labor, and HIV-risk behavior [at post-intervention follow-up for children ≥ age 16]).

#### Cost

We take a payer’s perspective and measure both direct and indirect costs for each sector, including those that have been paid for from grant funding, excluding research-related costs. Costs are allocated to their funding mechanism and each listed explicitly so that policy makers can assess both total costs and cost elements so that they can work with Education and Health to reduce or increase costs (understanding associated outcomes). Direct costs to the Education sector include teacher salaries, administrative salaries, supplies provided, salary of teachers who fill-in for the teachers providing the intervention, communication costs, transportation costs for trainings and supervision, and any incidentals that they may provide to caregivers or children. Direct costs to the Health sector are similar with the added cost of a stipend for the CHVs. Indirect costs to the Education and Health sectors that will need to be covered in the future are the costs of the salaries and benefits of local trainers, supervision, and the training costs including training time and venue rental.

#### Covariates

At baseline, we collect data on organizational, leader, and counselor characteristics that may affect intervention implementation and/or intervention effectiveness. At the organizational level, covariates include staffing (number of teachers or CHVs), size (number of children in the school; size of the community), and existing programs. Organizational information is mostly collected via interviews with the site leader (head teacher/deputy teacher or CHEW) using a measure designed for the purposes of this study. We also use standardized measures designed to capture organizational readiness [47] and implementation climate [48] that were reviewed for appropriateness by Kenyan partners and translated/back-translated to ensure construct validity. At the leadership-level, we assess general leadership [49] and implementation-specific leadership [50], also using standardized measures for which we followed similar procedures as the organizational measures. At the counselor-level, we capture demographics (e.g., age, sex), background characteristics (e.g., years in role, education), any training in, or past experience with mental health, and participation in supervision (e.g., dose). When possible, we also assess other implementation constructs using existing measures developed for use in low-resource contexts [51] and/or standardized measures reviewed and translated for appropriateness to assess intervention acceptability, feasibility [52], and perceived intervention effectiveness [51]. For theorized important constructs for which existing scales are not available or are not relevant (i.e., behavioral control, behavioral intentions, self-efficacy, appropriateness, innovation-values fit), we followed measure construction guidelines from the Theoretical Domains Framework [53, 54].

We also assess child, guardian, and community-level covariates that may impact implementation or intervention effectiveness. At the child- or guardian-level, these include child and guardian demographics (e.g., child age, sex, tribe) and background characteristics (e.g., child and guardian health, household composition, educational status, economic status, etc.). We assess several community-level characteristics (e.g., total number of orphans ages 11–14, main occupations in communities, perceived social mobility).

### Aim 1: identify actionable IPPs that predict adoption (delivery) and fidelity (high-quality delivery) after 10 sites in each sector implement TF-CBT

#### Aim 1 procedures

The goal of Aim 1 is to identify IPPs from the initial 10 schools and 10 communities in Sequence 1 (see Fig. 2) to both guide implementation support for subsequent sites (Aim 1a) and to generate testable hypotheses about IPPs that may predict implementation success, which will be tested in Aim 2 using data from all sites (40 schools; 40 villages). Following delivery of TF-CBT in Sequence 1 (10 sites in each sector), we conduct a case comparison analysis in which we identify 6 sites per sector (12 total) that represent unique aspects of the sites (e.g., urban vs. rural; higher/lower levels of leader support; student-teacher ratio) that might result in different ways in which sites successfully implement TF-CBT. Each site (school or village) is considered a case. Both qualitative and quantitative data are collected from counselors (*N* = 36) and leaders (*N* = 12) to understand barriers and facilitators to TF-CBT implementation and IPPs that are unique to each sector (Education vs. Health), overlapping across sectors, and those that may be related to site characteristics (e.g., rural location; small school).

#### Aim 1 analyses

We use a case comparison analysis, in which mixed methods are used to understand IPPs associated with successful implementation in Education and Health. A subset of successfully implementing sites (6/10 schools; 6/10 communities) are selected for heterogeniety on demographic and other characteristics (e.g., large school vs. small schools; urban vs. rural communities) to explore how implementation processes differ, such as the number and type of IPPs deployed as well as variation in how IPPs are deployed (e.g., whether leadership took different forms in different cases). We code data from interviews with counselors (teachers; CHVs) using a deductive approach [55] to identify IPPs from Weiner’s organizational theory of implementation effectiveness [26] and the literature [56]. Other IPPs that emerge during interview review are also incorporated into the codebook. All interviews are double-coded and discrepancies are reconciled through discussion to consensus [55]. In order to achieve convergence [55], qualitative data are examined alongside quantitative data on organizational, leader, and counselor-level constructs.

Following the case comparison analysis, the Ace Africa, University of Washington (UW), and Duke teams review and discuss findings. A priority is establishing straightforward, simple language for implementation constructs (e.g., implementation climate) that can be understood by all stakeholders. A 3-day BASIC Collaborative Team (BCT) meeting is then held with BASIC participants and stakeholders with the goal of collaboratively building plans for implementation facilitation for subsequent sequences. The BCT includes Ace Africa, UW, and Duke study members and local trainers, selected counselors and leaders from high-performing sites in Sequence 1, and other stakeholders (Ministry officials). The Ace Africa team presents results from Sequence 1 sites, orients the BCT to goals (provide future sites with support), and presents identified barriers and IPPs, including attention to variation by sector and site (urban/rural). Members split into Education and Health teams to discuss identified IPPs and generate additional ideas for actionable IPPs that would be practical in their setting. Ace Africa members facilitate this work, ensuring the focus remains on actionable IPPs (i.e., practical and feasible strategies; not those requiring new funding or resources). BCT members also collaborate on developing the appropriate timeline for implementation facilitation visits (i.e., frequency, timing), materials to guide facilitation (called "coaching" locally) for each visit, which IPPs should be prioritized (among those used in Sequence 1 sites), and which IPPs need to be addressed at which visits. By the end of the retreat, we have a list of IPPs for each sector from which sites could select IPPs for their own implementation planning, and a plan for facilitation visit frequency (6 meetings) and timing (e.g., before TF-CBT training, immediately post-training, early in delivery, planning for sustainment).

#### Implementation facilitation for new sites

A subset of BCT-attendees is selected as the initial implementation facilitators for sites in Sequences 2–7. One local Ace Africa trainer and 1 counselor from an experienced site (teacher or CHV) form a facilitation team that supports new sites in implementation. Leaders (head teachers, CHEWs) from experienced sites also are included in facilitation, but due to job demands, primarily connect with new leaders by phone (vs. in-person). During each visit, facilitators support new sites in developing tailored plans to support implementation using IPPs identified in the BCT.

### Aim 2. Identify causal conditions that explain implementation success in both sectors

The goal of Aim 2 is to identify causal conditions of adoption and fidelity, including hypothesized IPPs from Aim 1 (some targeted in implementation facilitation) and any additional IPPs that emerged during implementation in all sites (Aim 2a). We also identify causal conditions that predict sustainment in both sectors (Aim 2b). We examine sustainment for the subset followed for 2 years after the initial implementation year (20 in each sector). We expect that causal conditions that predict adoption, fidelity, and sustainment will include: 1) IPPs targeted by implementation facilitation (e.g., workload adjustment so counselors can provide TF-CBT), 2) other, non-targeted implementation factors from our conceptual model (e.g., organizational climate), and 3) additional important, but less actionable constructs (e.g., leadership turnover; delays in salary provision).

#### Aim 2 analyses

For Aim 2, we use generalized-set qualitative comparative analysis (QCA) [57]. QCA is a configurational approach that uses Boolean algebra to investigate logical relationships between causal conditions and an outcome of interest [58, 59]. QCA enables work with small samples [58, 60–64], and assumes causality is complex [58, 62, 65, 66]. This complexity occurs via *equifinality* (i.e., multiple causal pathways to same outcome), and/or *conjunctural causation* (i.e. pathways in which combinations of causal conditions result in the outcome of interest) [58]. QCA is particularly appropriate for investigating which causal conditions are *necessary,* and which combinations of causal conditions are *sufficient* to produce the outcome (e.g. acceptable fidelity) in small samples [58, 66]. Acceptable fidelity in some schools may require IPPs A and B, combined with specific individual-counselor characteristics, but other schools may require IPPs B and C (but not A).

For example, Table 4 illustrates the *necessity* of supervision (a causal condition present in all cases of acceptable fidelity), and the *sufficiency* of supervision with varying counselor characteristics (e.g., positive intention), otherl IPPs (e.g. workload adjustment and/or resources), and varying organizational context (e.g., positive organizational climate, and/or positive organizational readiness for change, and/or positive innovation-values fit). In Health, some combinations (“recipes”) may overlap with Education, while others may be unique.

**Table 4:** Examples of Possible Combinations of Constructs Resulting in High Fidelity

	Intention	Implementation Policies and Procedures				Organizational Context			High Fidelity
	Positive Intention	High Supervision	Leader Signals Support	Workload Adjustment	Resources	High Org. Readiness	Positive Implementation Climate	Positive Innovation Values Fit	
Case 1	No	Yes	Yes	Yes	Yes	Yes	Yes	No	Yes
Case 2	Yes	Yes	Yes	No	Yes	Yes	Yes	Yes	Yes
Case 3	No	Yes	No	Yes	Yes	No	Yes	Yes	Yes
Case 4	Yes	Yes	No	Yes	Yes	Yes	No	No	Yes

We will investigate necessary and sufficient causal conditions and pathways that explain adoption, fidelity, and sustainment of TF-CBT using Boolean minimization [67] procedures. We follow the QCA procedural protocol outlined by Thiem [68] for elimination of redundant causal conditions (variables) by transforming the raw data into a data matrix (truth table), minimization of the truth table to a prime implicant (PI) chart, and decomposition of the PI chart to investigate necessary causal conditions. The minimum number of cases required to identify a set of conditions that lead to the outcome of interest will be set to 1 to maximize *inclusiveness* [58]. We will evaluate *coverage*, how much of the outcome is explained by each causal pathway and the solution term, and *consistency*, the degree to which sites exhibiting a specific combination of conditions also exhibit the outcome of interest, as outlined by Schneider and Wagemann [69]. We will report on model ambiguity [70, 71] and present empirical results using the parsimonious solution type to avoid the potential for reporting results that claim causal relationships with no evidence [72].

### Aim 3*.* Test TF-CBT effectiveness (child mental health, functioning) and cost in both sectors

Results from Aim 3 will be needed to influence policy changes. Understanding TF-CBT’s effectiveness when delivered in Health and Education is important. Knowing the cost in Education and Health is critical for decision-making of interested government sectors to continue TF-CBT scale-up throughout the county and country. We select a cost accounting and comparison approach due to its flexibility, ease of use, ability to easily identify item-by-item costs and change the outcome of interest as desired by local policy makers and implementers. We measure costs of the intervention for improvement in children’s mental health outcomes. We will also provide the costs of the intervention as implemented by teachers and CHVs with the societal benefits of school retention, passage of the exam to enter secondary school, and fewer high-risk sexual activities. In low-resource settings, even seemingly small differences in cost are important.

#### Aim 3 analyses: TF-CBT effectiveness

A combination of within-cluster within-child and between-cluster between-child data is used to assess intervention effectiveness on child outcomes. We will examine theoretically relevant covariates and, importantly, include implementation outcomes from Aim 2 (i.e., adoption, fidelity). For statistical models, we will use a stepped-wedge analytic approach that leverages the staged scale-up of TF-CBT [73]. Continuous child outcomes will be assessed using linear mixed models. Dichotomous child outcomes will be assessed using generalized linear mixed models with a log or logit link for relative effects and with an identity link for absolute effects if models converge. We will analyze the unit increment or decrement (or probability for dichotomous measures) of an outcome (e.g. PTS) at a given time, with fixed effects for time and intervention status and random effects for cluster and child. All analyses are intent-to-treat. Because there are 2 different sectors for delivery (Education and Health) and a comparison condition, intervention status is a 3-level factor to be modeled as 2 dummy variables. We will evaluate change in pairwise correlation of within-child repeated measures over time, as well as pairwise correlations between different children in the same cluster at different time points. We will include random slopes for interaction of child with time or of cluster with time*,* as appropriate [74, 75]. Correct correlation structure specification is important to avoid bias in estimated effects [74, 75]. Additional analyses will explore potentially relevant interactions that may indicate effects within sector.

#### Handling missing data

We will use available data to examine patterns and predictors of missingness both by sequence and condition to assess potential for biased estimated treatment effects. Because likelihood-based modeling approaches are valid under the missing at random assumption, including the situation where predictors measured at time of enrollment are predictive of missing outcomes, our primary analytic approach can provide valid inference if covariates predictive of missingness are included in the model. We will perform sensitivity analyses that adjust for those covariates. If we identify evidence that the missing at random assumption is violated, alternative pattern mixture approaches will be used as sensitivity analyses [76].

#### Power/sample size calculation

The SW-CRT design may be more efficient than other cluster trials, particularly when intracluster correlation coefficients (ICC) are high [77, 78]. To assess sensitivity to underlying assumptions, we used the *Shiny CRT Calculator* [79] to calculate power for standardized effect sizes for an incomplete SW-CRT cohort design with 40 clusters (12 youth per sector per cluster) randomly assigned to 7 sequences under 3 different correlation structures. Detailed specifications and assumptions are included in Additional file 2 [79–83]. The power to detect effect sizes as low as .25 at the 2-tailed .05 level of significance was estimated to be 99% assuming an exchangeable correlation structure, 95% assuming 2-period decay, and 92% assuming discrete time decay. Study power is very good, even with the most flexible model (discrete time decay).

#### Aim 3 analyses: cost accounting

Direct and indirect costs of TF-CBT will be collected systematically from schools (e.g., salaries, fringe, materials provided, room costs, teacher substitution for missed classes, etc.), CHEWs, CHVs, and Ace Africa (e.g., air time, supervisor salary and fringe, transportation costs, etc.). Costs will be summarized by category to allow policy makers and future implementers to easily identify what costs can be altered or might vary depending on context.

Detailed data management procedures, composition of the data monitoring committee, unintended effects, procedures for auditing trial conduct and communicating protocol modifications, model consent forms, and questionnaires can be found in our IRB protocols.

### Dissemination

Sharing early findings with participants, ministry leaders and other stakeholders is built into the design of BASIC with the BCTs and implementation facilitation. In addition, communications via reports and in-person discussions with ministry leaders, community leaders, site leaders, and participants enable audience-specific dissemination of findings. Peer-reviewed publications and conference abstracts will adhere to journal and professional standards for authorship and will include all relevant collaborators. The data are sensitive in nature and may not be sufficiently “de-identifiable” as to remain useful while maintaining confidentiality. To the extent data are de-identifiable, we will make data available in accordance with IRB and National Institutes of Health guidance.

## Discussion

This study is the next step in our research agenda of testing the effectiveness of EBTs in successive stages that systematically increase local expertise and responsibility and decrease external expert involvement. The goal is to learn what makes an “enabling” context—that is, what policies and practices are necessary for supporting the delivery of mental health interventions. Our ultimate goal is to enhance knowledge around effective, feasible, and sustainable implementation strategies in low-resource contexts globally. We leverage the increasing body of evidence supporting community-based, collaborative approaches to provide implementation support, given their substantial promise for local ownership, oversight, and fit with the cultural context. These approaches offer a potentially sustainable avenue in low-resource settings where budgets are constrained and monetary incentives are low. To our knowledge, this is the first study to examine questions related to scale-up and sustainability of a child-focused EBT in both Education and Health, and one of few global implementation studies that includes an organizational focus and a focus on sustainment. In a sustainability review of health interventions in Sub-Saharan Africa, *none* focused on mental health and most focused on *what* was sustained, not *by whom or how* [41].

The rollout of TF-CBT in Education and Health to date has been welcomed, with all sites expressing interest in TF-CBT training and adoption. From early qualitative interviews and responses on quantitative scales from the first 10 clusters beginning implementation in Sequence 1, teachers and CHVs trained in TF-CBT seem to find the intervention acceptable, believe it will have benefits for children, and have noted the applicability of the skills for their own lives and community. We have observed inter-ministerial collaboration that appears to support implementation, with ministries working together to provide TF-CBT to their community (e.g., schools offering classrooms to CHVs for holding groups; CHVs supporting teachers with outreach to guardians in the community). Ace Africa supervisors have creatively used technology to facilitate supervision and lessen the need and cost of in-person supervision by using short message service (SMS) and WhatsApp to communicate, and sometimes to review counselors’ practice (e.g., reviewing an audio recording of counselors’ practice role play, uploaded using WhatsApp). The implementation facilitation teams initiated facilitation smoothly after the BCT, and Ace Africa supervisors anecdotally report that the facilitation intervention, initiated for Sequence 2 clusters, seems to be beneficial.

However, there have been practical challenges, including higher than expected costs for a few implementation activities. Hosting training for teachers and CHVs (training venue, catering, transportation reimbursements) has been more expensive than expected. Transportation for supervisors to meet with their counselors in-person on occasion, and to observe groups, has also been costly. Due to our focus on children who are parentally-bereaved, small schools with fewer students do not always have as many orphaned children in need of mental health care and groups may consist of fewer than 8 children, lowering the overall child sample in our study. Finally, while expected from the literature and our own experience [15], guardian attendance at groups has been more inconsistent than child attendance, given that many guardians are busy with livelihood-generating activities, and some send children to schools that are not their village school (if another school has better academic performance). Thus, guardians may not be in close walking distance to schools, hindering participation in TF-CBT sessions. To engage guardians, counselors often do more outreach and provide individual make-up sessions for guardians. While this addresses the clinical challenge, it adds burden to counselors, increasing the time they spend delivering TF-CBT.

The potential promise of task-sharing for closing the mental health treatment gap goes unrealized if evidence-based guidance to inform scale-up and sustainment is unavailable. Results from the BASIC trial should provide information that will be beneficial for organizations, their leaders, ministry officials, and policy makers looking to implement mental health therapies into existing systems. Our focus on identifying policies and practices that support implementation in two different sectors within a low-resource context should generate pragmatic guidance for how to support mental health service delivery that could be broadly applicable.

### Trial status

At the time of manuscript submission (October 2019), the study is ongoing; we are in the early stages of data collection. We have enrolled children and guardians for Sequences 1–3 (20 of 40 clusters), as well as counselors and site leaders for all sequences. Additionally, implementation facilitation for Sequences 2 and 3 have begun; Sequence 4 will commence in January 2020.

## Supplementary information

**Additional file 1.** SPIRIT protocol checklist.

**Additional file 2.** Specifications of power calculations.

## Data Availability

The data generated from this study will be available from the corresponding author on reasonable request.

## Abbreviations

BASIC: Building and Sustaining Interventions for Children
BCT(s): BASIC Collaborative Teams
CHEW(s): Community health extension workers
CHV(s): Community health volunteers
CONSORT: Consolidated Standards of Reporting Trials
EBT(s): Evidence-based treatments
HIV: Human Immunodeficiency Virus
ICC: Intracluster correlation coefficient
IPP(s): Implementation policies and practices
IRB: Institutional Review Board
KEMRI: Kenya Medical Research Institute
LMIC(s): Low- and middle-income countries
MPI(s): Multiple Principle Investigators
NIMH: National Institutes of Mental Health
PI: Prime implicant
PTS: Posttraumatic stress
QCA: Qualitative comparative analysis
RCT(s): Randomized controlled trials
RDAC: Research Design and Analysis Core
SMS: Short message service
SPIRIT: Standard Protocol Items: Recommendations for Interventional Trials
SW-CRT: Stepped wedge, cluster randomized controlled trial
TF-CB: Trauma-focused Cognitive Behavioral Therapy
TTT: Train-the-Trainer
UNICEF: United Nations Children Fund
US: United States
UW: University of Washington
WHO: World Health Organization
