# Supplementary material for: Advancing successful implementation of task-shifted mental health care in low-resource settings (BASIC): protocol for a stepped wedge cluster randomized trial
Source: BMC Psychiatry. 2020 Jan 8;20:10. doi: 10.1186/s12888-019-2364-4 (PMC6947833; doi:10.1186/s12888-019-2364-4)
Supplement: Supplementary file 2 — Additional file 2. Specifications of power calculations. [file 12888_2019_2364_MOESM2_ESM.docx]

**Power Analysis Details**

The SW-CRT design may be more efficient than other cluster trials, particularly when the intracluster correlation coefficient (ICC) is high, as expected here given the rural and urban areas.^77,78^ In order to assess sensitivity to underlying assumptions, we calculated power for standardized effect sizes for an incomplete SW-CRT cohort design under three different correlation structures. Up to three different pairwise correlations are considered in these structures, namely the within-period ICC (WP-ICC: two different people in the same cluster at the same time point), the between-period ICC (BP-ICC: two different people in the same cluster at different time points), and the within-person ICC (the same person at two different time points). Those three correlation structures allow for different relationships between the within-period and between-period ICC, with the within-person separately specified and, in our case, assumed constant over time and the same for all three scenarios considered. The three structures are given as follows, ordered from the least to most flexible: (1) exchangeable^80^ (WP-ICC=BP-ICC), (2) two-period decay (WP-ICC and BP-ICC are different and the BP-ICC is constant irrespective of time between periods)^81,82^ and (3) discrete-time decay^83^ (WP-ICC and BP-ICC are different and the BP-ICC decays exponentially with increasing distance between time periods).^81,82^ All approaches are based on an underlying linear mixed model and calculations were performed using the *Shiny CRT Calculator*^79^ by supplying the appropriate design matrix. We assumed 40 clusters were randomly assigned to 7 sequences (with 10 to the first and 5 to each of the other six sequences) under the incomplete SW design shown in Figure 1 with 12 youth per sector in each cluster (target is 8 girls and 8 boys per sector per cluster) and a coefficient of variation of cluster size of 0.23 (based on preliminary field data). For all three correlation structures, we assumed a within-person correlation of 0.6 (assumed constant over time). Under the exchangeable model, the BP-ICC and WP-ICC were assumed to both be 0.1 with no decay over time. For the two-period decay model, the between BP-ICC was assumed to be lower than the WP-ICC at 0.06 (and to be constant over time). Those same two parameters were assumed in the discrete-time decay model, where as noted above, the BP-ICC is assumed to decay exponentially once measurements were more than 2 periods apart. The power to detect effect sizes as low as .25 at the two-tailed .05 level of significance was estimated to be 99% assuming an exchangeable correlation structure, 95% assuming two-period decay, and, 92% assuming discrete time decay. Study power is very good, even with the most flexible model (i.e. discrete time decay).
